# Modified cetyltrimethylammonium bromide DNA extraction for *Mycobacterium tuberculosis* whole genome sequencing

**DOI:** 10.11604/pamj.2026.53.28.50239

**Published:** 2026-01-22

**Authors:** Susan Musau, Susan Odera, Noel Onyango, Victor Moses Musyoki, Steve Wandiga, Videlis Nduba, Marianne Mureithi

**Affiliations:** 1 Department of Medical Microbiology and Immunology, University of Nairobi, Nairobi, Kenya,; 2 Tuberculosis and HIV Co-Infection Training Program, Nairobi, Kenya,; 3 Kenya Medical Research Institute (KEMRI), Nairobi, Kenya

**Keywords:** Cetyltrimethylammonium bromide, deoxyribonucleic acid, *Mycobacterium tuberculosis* isolates, resource-limited countries, Kenya

## Abstract

Efficient extraction of high-quality genomic deoxyribonucleic acid (DNA) from Mycobacterium tuberculosis (MTB) is a critical step for whole genome sequencing (WGS) and downstream molecular applications. However, the lipid-rich MTB cell wall continues to limit DNA recovery, and existing protocols do not consistently address this challenge. This study aimed to refine the cetyltrimethylammonium bromide (CTAB)-based DNA extraction protocol to achieve improved DNA yield and purity for downstream WGS applications. We conducted a cross-sectional laboratory-based protocol refinement study nested within an ongoing MTBC genetic diversity and drug resistance study in western Kenya, using archived Mycobacterium Growth Indicator Tube (MGIT) cultured isolates. The entire MGIT broth from the MTBC-positive isolates was centrifuged before extraction to maximize bacterial biomass recovery, followed by an extended lysozyme incubation to promote enzymatic cell wall disruption. The process combined enzymatic lysis, mechanical disruption, CTAB-based purification, and isopropanol precipitation. Deoxyribonucleic acid concentration and purity were determined by NanoDrop spectrophotometry. A total of 325 MTB isolates were processed. Deoxyribonucleic acid (DNA) concentrations ranged from 5.41 to 2,052.60 ng/μL (median: 65.21 ng/μL), with A260/280 purity ratios between 1.36 and 2.08. 60% of samples achieved DNA concentrations ≥50 ng/μL, and overall, 89% (290/325) of the samples successfully passed sequencing quality control. The majority of the samples were successfully sequenced. The refined CTAB protocol effectively overcomes the challenge posed by the MTB cell wall, yielding high-quality DNA suitable for WGS. It provides an accessible and scalable approach for laboratories in high TB burden and resource-limited settings.

## Introduction

Tuberculosis remains a major global health concern and was the leading cause of death from a single infectious agent before the COVID-19 pandemic [1]. Kenya is among the 30 high TB burden countries, with higher prevalence reported in the western region [2]. The WHO End TB Strategy aims to reduce TB incidence by 90% and mortality by 95% by 2035, emphasizing the need for rapid, accurate diagnostics and timely, effective treatment [3]. *Mycobacterium tuberculosis* is characterized by slow growth and a lipid-rich cell wall that confers resistance to standard lysis methods [4]. Although culture is the diagnostic gold standard, liquid culture requires additional downstream testing, and the growing use of WGS for TB surveillance is constrained by difficulties in extracting high-quality DNA due to the mycobacterial cell wall [5,6].

Cetyltrimethylammonium bromide (CTAB)-based DNA extraction methods are widely used for their effectiveness and accessibility, but when applied to MGIT-cultured clinical isolates, methodological variability leads to inconsistent DNA yield and purity [4,7]. Studies employing short lysozyme incubation or partial culture volumes have frequently reported low DNA yields, whereas extended enzymatic lysis has been associated with improved DNA yield and quality, mainly for solid culture media [8,9]. Evidence for optimal CTAB-based extraction from MGIT-cultured isolates remains limited and inconsistent [10]. This study aimed to refine a CTAB-based DNA extraction protocol to improve DNA yield and purity from MGIT-cultured MTB isolates, hypothesizing that improved cell wall disruption and optimized purification would enhance suitability for downstream whole-genome sequencing.

## Methods

**Study design:** this was a cross-sectional, laboratory-based protocol refinement study nested within an ongoing MTBC genetic diversity and drug resistance study, using archived MGIT-positive isolates collected in western Kenya (2021-2022). A total of 325 isolates were selected from 1,053 using consecutive and stratified random sampling, revived in June 2024, and analyzed with a focus solely on refining the CTAB-based DNA extraction step through targeted modifications to improve biomass recovery and DNA quality.

**Reagent preparation and safety considerations:** reagents were prepared using standard procedures. Lysozyme (Roth & Co., USA; 10 mg/ml) and Proteinase K (Qiagen, Germany; 10 mg/ml) were prepared in DEPC-treated water (Invitrogen, USA), while SDS (Invitrogen, USA; 10%) was prepared in distilled water. Sodium chloride (Thermo Fisher Scientific Inc., USA; 5 M) and CTAB-NaCl buffer (CTAB: Merck, Germany) were prepared with heating to ensure complete dissolution. Chloroform: isoamyl alcohol (24:1; Thermo Fisher Scientific Inc., USA/Acrylis, USA) and 70% ethanol solutions were prepared and stored as specified. All buffers were prepared using distilled water according to manufacturers´ instructions, and standard laboratory safety procedures, including the use of personal protective equipment and biosafety cabinets, were followed.

**Deoxyribonucleic acid extraction procedure:** mycobacterial strains were cultured in MGIT media for 2-3 weeks and visually examined for contamination before extraction. The whole MGIT growth was transferred into 15 ml Falcon tubes and centrifuged for 20 minutes at 3,000 rpm at room temperature. After discarding the supernatant, the resulting pellets were resuspended in 1 ml of 1X TE buffer, transferred to 2 ml screw-cap tubes, and centrifuged at 10,000 g for 10 minutes at room temperature. After discarding the supernatant, 400μl of TE buffer was added, and the cells were heat-killed in a 95°C water bath for 30 minutes. The suspension was briefly centrifuged, followed by the addition of 50μl lysozyme (10μmg/ml) and incubation at 37°C overnight.

Isopropanol and 70% ethanol were maintained at 4°C and -20°C, respectively, while the CTAB-NaCl buffer was pre-warmed to 60°C. A freshly prepared SDS/Proteinase K mixture was added to each sample and incubated at 65°C, followed by the addition of NaCl and pre-warmed CTAB-NaCl with further incubation. Chloroform: isoamyl alcohol (24: 1) was then added, and samples were vortexed and centrifuged at 14,000 g. The resulting pellet was washed with prechilled 70% ethanol and centrifuged to complete DNA purification. The supernatant was carefully removed, and residual liquid was eliminated without disturbing the DNA pellet, followed by an additional centrifugation and air-drying of the pellet at 60°C. Deoxyribonucleic acid was rehydrated in 40 μl of 1x TE buffer, stored at 4-8°C overnight, and subsequently at -20°C before shipment. Deoxyribonucleic acid (DNA) concentration and quality were assessed using NanoDrop spectrophotometry.

**Quality control procedures:** deoxyribonucleic acid extractions were done in multiple standardized batches with negative controls and routine MGIT contamination checks; DNA concentration and purity were measured by NanoDrop, suboptimal samples were re-extracted, and no systematic batch-related deviations were observed. Post hoc WGS suitability benchmarks (≥50 ng/μL and A260/A280 ≥1.7) were used for contextual assessment, but all DNA samples were submitted for WGS with no exclusions based on these metrics.

**Statistical analysis:** data were analyzed using R, with DNA yield and purity summarized descriptively using proportions, ranges, and medians; normality was assessed using the Shapiro-Wilk test, and no inferential statistics, power, or effect size calculations were performed due to the study´s exploratory, methodological design.

**Ethical approval:** ethical approval was obtained from the Kenyatta National Hospital-University of Nairobi Ethics and Research Committee (P4/01/2024) and the Kenya Medical Research Institute Scientific and Ethics Review Unit (KEMRI/SERU/CRDR/XXX/4918), with study authorization granted by the National Commission for Science, Technology and Innovation (NACOSTI/P/24/35978). Institutional permission was obtained from the Kenya Medical Research Institute-Centre for Global Health Research and the TB Branch in Kisumu.

## Results

Deoxyribonucleic acid extracted from 325 archived *Mycobacterium tuberculosis* isolates using the modified CTAB protocol showed wide yield variation (5.41-2,052.60 ng/μL), with a median concentration of 65.21 ng/μL and an IQR of 32.57-119.83 ng/μL (Table 1). Many samples exceeding 50 ng/μL were consistent with prior CTAB-based studies [8]. Deoxyribonucleic acid purity (A260/A280) ranged from 1.36 to 2.08, with most values between 1.5 and 1.75 and a mean of ∼1.63, while only 25% fell below 1.55, indicating consistent performance [4]. All 325 samples were submitted for WGS, of which 290 (89.2%) passed sequencing quality control and generated usable data.

**Table 1:** mean and median of nucleic acid (ng/μL) & A260/A280 ratio

	Observations	Mean	Minimum	Maximum	25%	50%	75%	No. (%) meeting pragmatic threshold	Overall sequencing success rate
Nucleic acid (ng/μL)	325	109.35	5.41	2052.6	32.57	65.21	119.83	196 (60%) ≥50 ng/μL	290/325 (≈89%)
A260/A280 ratio	325	1.63	1.36	2.08	1.55	1.64	1.70	147 (45%) ≥1.7	

Sequencing thresholds were used for descriptive purposes only; all samples were sequenced regardless of concentration or purity

## Discussion

This study addressed the need for improved TB diagnostics by refining a CTAB-based DNA extraction protocol through whole-culture MGIT centrifugation and extended overnight lysozyme incubation to enhance cell wall disruption and DNA recovery. Consistent with previous studies, extended enzymatic lysis was associated with improved DNA yield and acceptable purity [7,8], whereas shorter incubation periods generally resulted in low DNA concentrations despite comparable purity [4,9]. Mechanical disruption has been shown to compensate for shorter lysis in select settings [10], further underscoring the importance of effective cell wall disruption. Despite some variability in purity (A260/A280: 1.35-2.08), most samples met acceptable standards [4], and such variability is unlikely to affect downstream sequencing performance [7].

The refined CTAB protocol yielded DNA suitable for both short- and long-read sequencing, with most samples passing sequencing quality control, while remaining variability likely reflects MGIT culture and batch-related factors rather than extraction efficiency alone. Owing to its cost-effectiveness, scalability, and adaptability, the protocol is well-suited for processing MGIT-cultured clinical isolates in resource-limited settings [7]. Despite being labor-intensive, its flexibility for optimization aligns with the needs of laboratories conducting TB genomic surveillance and molecular epidemiology in high-burden regions [4], supporting the reliable generation of high-quality genomic data for accurate strain characterization

**Limitations:** this study was limited by the absence of direct comparisons with standard CTAB methods or commercial kits, evaluation in a single laboratory, and reliance on descriptive analyses and NanoDrop-based DNA quality assessment. Applicability beyond MGIT-cultured MTB isolates remains uncertain; however, consistent performance across multiple batches supports the protocol´s practical utility in routine, resource-limited laboratory settings.

## Conclusion

This study demonstrates that the refined CTAB-based DNA extraction protocol reliably produces genomic DNA suitable for whole-genome sequencing from MGIT-culture MTB isolates. With 290 of 325 samples (∼89%) passing sequencing quality control, the protocol showed consistent performance across batches and provides a cost-effective, accessible solution for TB genomic applications in resource-limited, high-burden settings.

### What is known about this topic

- Cetyltrimethylammonium bromide-based DNA extraction is widely used for MTB but is time-intensive and yields variable DNA quality across laboratories;
- Commercial extraction kits simplify workflows but are costly and difficult to sustain in resource-limited settings;
- Whole genome sequencing requires high-quality DNA, which many low-resource laboratories struggle to obtain consistently.

### What this study adds

- The modified CTAB protocol yields high-quality MTB DNA suitable for whole-genome sequencing, supporting accessible molecular surveillance;
- The protocol provides a detailed stepwise procedure for CTAB DNA extraction with high replicability.

## References

[1] World Health Organization (2025). Global tuberculosis report 2024.

[2] Enos M, Sitienei J, Ong´ang´o J, Mungai B, Kamene M, Wambugu J (2018). Kenya tuberculosis prevalence survey 2016: challenges and opportunities of ending TB in Kenya. PloS One.

[3] World Health Organization (2015). The End TB Strategy.

[4] Ndhlovu V, Mandala W, Sloan D, Kamdolozi M, Caws M, Davies G (2018). Evaluation of the efficacy of two methods for direct extraction of DNA from Mycobacterium tuberculosis sputum. J Infect Dev Ctries.

[5] World Health Organization (2007). Use of Liquid TB Culture and Drug Susceptibility Testing (DST) in Low and Medium Income Settings. Summary report of the Expert Group Meeting on the use of liquid culture media.

[6] Arslan N, Demiray-Gurbuz E, Özkütük N, Esen N, Özkütük AA (2024). Comparison of Four Different DNA Isolation Methods from MGIT Culture for Long-Read Whole Genome Sequencing of Mycobacterium tuberculosis. Jundishapur J Microbiol.

[7] Mogashoa T, Loubser J, Choga OT, Ngom JT, Choga WT, Mbulawa MB (2025). Whole genomic analysis uncovers high genetic diversity of rifampicin-resistant Mycobacterium tuberculosis strains in Botswana. Front Microbiol.

[8] Makamure B (2023). Assessment of Quantity and Quality of Multidrug-Resistant Tuberculosis DNA Extracts Stored at Different Temperatures. J Microbiol Infect Dis.

[9] Mohammadi S, Esfahani BN, Moghim S, Mirhendi H, Zaniani FR, Safaei HG (2017). Optimal DNA isolation method for detection of nontuberculous mycobacteria by polymerase chain reaction. Adv Biomed Res.

[10] Elton L, Aydin A, Stoker N, Rofael S, Wildner LM, Abdul JBPAA (2025). A pragmatic pipeline for drug resistance and lineage identification in Mycobacterium tuberculosis using whole genome sequencing. PLOS Glob Public Health.

